# Leiomyoma – a rare benign tumor of the female urethra: a case report

**DOI:** 10.1186/1752-1947-8-366

**Published:** 2014-11-13

**Authors:** Mário Maciel de Lima Junior, Cleyton Barbosa Sampaio, José Geraldo Ticianeli, Mário Maciel de Lima, Fabiana Granja

**Affiliations:** 1 Department of Uro-Gynecology of Reference Center of Women’s Health Hospital, Roraima, Brazil; 2 Cathedral College, Roraima, Brazil; 3 Biodiversity Research Center, Federal University of Roraima (CBio/UFRR), Roraima, Brazil

**Keywords:** Benign, Distal, Leiomyoma, Smooth muscle, Tumor, Urethra

## Abstract

**Introduction:**

Leiomyoma in the urethra is a rare occurrence. These are rare benign mesenchymal tumors that arise from the smooth muscle of the urethra. Such tumors often appear in females during their reproductive age (from menarche to menopause); the mean age of their appearance is approximately 41 years.

**Case presentation:**

We report here a case of a 52-year-old White woman who presented with complaints of sporadic hematuria, dyspareunia, and feeling of nodulation in her vagina.

**Conclusions:**

Histopathological studies confirmed the urethral leiomyoma, and the surgery completely resolved the original symptoms. Although the average age of occurrence of such tumors in females is about 41 years, the present case involves an older woman of 52 years. Most importantly, the mass was located in the distal urethra, an uncommon site of presentation of leiomyoma in females.

## Introduction

Leiomyomas are benign tumors of the smooth muscles. Although the tumors tend to be relatively common in the genitourinary and gastrointestinal tracts, the occurrences are less frequent in the skin and rare in the deep tissues. In general, soft tissue leiomyomas cause little morbidity. Leiomyomas can be classified under three categories: (i) cutaneous leiomyoma (leiomyoma cutis), (ii) angiomyomas (vascular leiomyomas), and (iii) leiomyomas of deep soft tissue [1]. Urethral leiomyomas are, in fact, classified under leiomyomas of deep soft tissue and are rare benign mesenchymal tumors that originate from the smooth muscle of the urethra [2-4]. Such a tumor was first described by Buttner in 1894. Although mostly observed in females of childbearing age, no age or gender is exempt [5]. These tumors present with a variety of symptoms including recurrent urinary tract infections (64.3%), urinary retention, heaviness due to tumor, voiding dysfunctions, and dysuria. A protruding mass from the external urethral meatus is common when the tumor is located in the distal part of the urethra [1,6,7]. There has been no report of any malignant transformation of this tumor [1,2,5].

Here we report a case of a distal urethral leiomyoma in a 52-year-old White woman who presented with complaints of sporadic hematuria, dyspareunia, and feeling of nodulation in her vagina. The diagnosis was confirmed on histopathology, and surgery completely resolved the original symptoms.

## Case presentation

**Figure 1:**
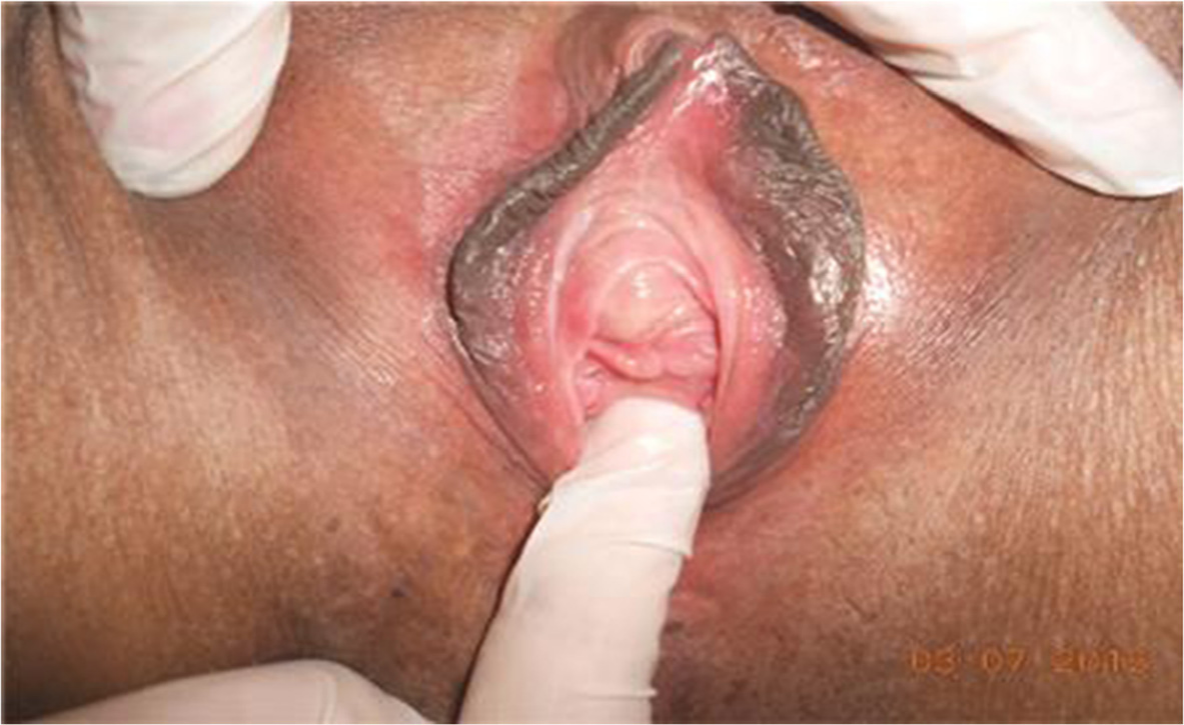
Local examination revealed a 2×2cm growth tumor arising from the urethra; examination was done during the surgical procedure.

A 52-year-old White woman presented with complaints of sporadic hematuria, dyspareunia, and feeling of nodulation in her vagina for the last 2 years. Her medical history revealed that she had two caesarean deliveries in the past, but she had no chronic disease.On physical examination, we detected a 2×2cm painless hard tumor without signs of inflammation at the level of the urethral meatus (Figure 1). Her urethroscopy results were normal. Flexible cystoscopy showed tumor in her distal urethra at the level between 3 o’clock and 6 o’clock position.

**Figure 2:**
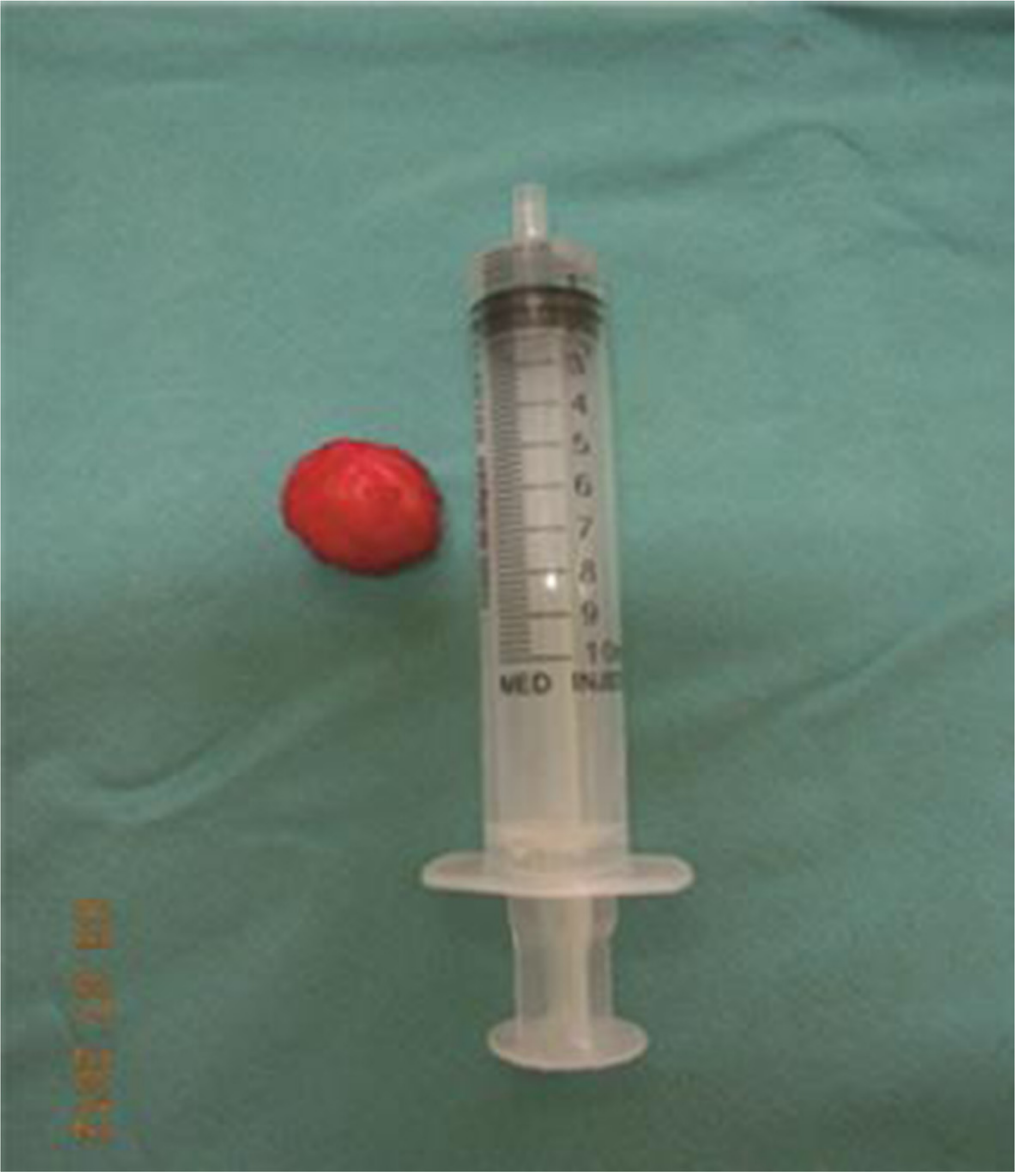
Surgical specimen (2×2cm) after procedure.

On confirming the position, she was operated under spinal anesthesia. The tumor was resected with suburethral incision (U-shaped inverted). The urethra was calibrated with an 18-French Foley catheter, and an indwelling Foley’s catheter was kept inserted for 10 days. She was discharged from the hospital on the second day.Histopathological study of the tumor revealed spindle-shaped smooth muscle fibers in a whirling pattern (Figure 2), confirming the diagnosis of leiomyoma.

The Foley catheter was removed on the 10th day. Her postoperative recovery was uneventful. She was symptom-free at the time of her last follow-up; complaints like fistula, dyspareunia, infection, and incontinence were not reported.

## Discussion

From among all the leiomyoma cases reported to date, the most common site of occurrence has been in the genital tract (95%), while the remainder are scattered over various sites, including the skin (230 cases), gastrointestinal tract (67 cases), and bladder (five cases) [8]. Although leiomyomas are very common in organs such as the uterus, the presentation of a urethral leiomyoma, as found in the present case, is very rare. Categorized as deep tissue leiomyomas, urethral leiomyomas are much larger than their superficial counterparts, and usually display a greater spectrum of histological changes; therefore, it is important to clearly distinguish them from leiomyosarcomas, which are statistically more common in deep soft tissue.

Benign neoplasms of the urethra may arise from any of its histological elements: transitional epithelium, stratified squamous epithelium, glandular epithelium, and smooth and striated muscle.

Leiomyoma of the urethra is a rare benign mesenchymal tumor that affects women more often than men [2-4]. It can affect the distal urethra, but the proximal segment is the most common site [4]. The tumor often appears during the reproductive age (from menarche to menopause) of women; the mean age in most cases is around 41 years [1,2,5]. The tumor has been reported to enlarge during pregnancy and shrink after delivery, suggesting a possible hormonal dependence [7]. In men, such tumors may originate from any part of the urinary tract, including the kidney, which is reported to be the most common site. Other sites of origin include the urinary bladder, prostate, scrotum, penis, spermatic cord, epididymis, and seminal vesicles. The site of the lesion determines the clinical features and therapeutic approach.

Diagnosis of such tumors is primarily based on clinical history, physical examination, and imaging techniques. A careful clinical examination, urethroscopy, and radiological examination of the lower urinary tract are essential in order to distinguish the tumor from urethrocele, urethral diverticulum, and caruncle. Most importantly, the tumor must be differentiated from its malignant counterpart, the leiomyosarcoma, which shows marked pleomorphism, increased cellularity, and frequent mitoses. In females, the tumor leiomyoma originates from the genital tract, excluding the uterus. The final diagnosis should be made based on the histopathological report. The most appropriate treatment for this condition is local surgical excision or transurethral resection [8,9]. There is usually no recurrence or malignant transformation after treatment. Leiomyomas do not recur or metastasize with the exception of the rare metastasizing leiomyomas of the uterus, and there are no reasons to believe that urethral leiomyomas behave in a different way [10]. No urethral leiomyoma has been reported to recur or metastasize to date.

In the present case, the patient belonged to the 50- to 59-year-old age group. The tumor typically affects the bulbous part of the urethra (80% of cases); however, the distal segment can also be affected, as found to occur in the current case, an uncommon site for leiomyoma in females.

After preoperative evaluation based on careful physical and histological examinations, leiomyomas are treated by excision, either by transurethral or open resection. In our case, the mass was located in the distal urethra, which is not the common site of presentation of leiomyoma in females. The tumor was successfully removed by resection with complete relief of her symptoms. Postoperative recovery was uneventful; subsequent complications, such as fistula, dyspareunia, infection, and incontinence were not reported.

## Conclusions

The present case of 52-year-old White woman with urethral leiomyoma is a rare case for two reasons. Firstly, the patient was older than the average age (41 years) of occurrences of such tumors. Secondly, the position of the tumor makes it a very interesting case; the mass was located in the distal urethra, an uncommon site of presentation of urethral leiomyoma in females. Her uneventful postoperative recovery without any complication suggests surgery is the best option to manage such tumors. Hence, we recommend resection of such tumors as the best possible management.

## Consent

Written informed consent was obtained from the patient for publication of this case report and accompanying images. A copy of the written consent is available for review by the Editor-in-Chief of this journal.

## Competing interests

The authors declare that they have no competing interests.

## Authors’ contributions

MML Jr was the major contributor in writing the manuscript, and performed the surgery. CBS analyzed and interpreted the patient data, and performed the surgery. JGT analyzed and interpreted the patient data. MML performed the surgery. FG analyzed and interpreted the patient data. All authors read and approved the final manuscript.
